# Supplementary material for: Proactive Contact Tracing
Source: PLOS Digit Health. 2023 Mar 13;2(3):e0000199. doi: 10.1371/journal.pdig.0000199 (PMC10010527; doi:10.1371/journal.pdig.0000199)
Supplement: S1 Appendix — Discussion on possible designs of PCT based applications. (PDF) [file pdig.0000199.s001.pdf]

S0 Appendix: Decentralized and Centralized versions of PCT

From a privacy perspective, we can view the architecture of the PCT smartphone application as three components:

- **On-device execution of the risk estimation model**: A model (e.g., a set of rules) takes in various features as inputs and outputs risk estimates for that user. The features are of two types - (a) locally available on the smartphone, e.g., symptoms, pre-existing conditions, test results, etc., that are used only with the user's consent. This information never leaves the smartphone, mitigating the risk of leaking personally identifiable information. (b) risk messages from past encounters - the smartphone app manages to send and receive these risk messages according to the network protocol. We discuss this in the third point below.
- **Transmission of the risk estimation model to the device**: The model used for risk estimation is broadcasted to the users at a predetermined frequency. For example, it may be desirable to update the rules every two weeks, in which case a centralised server would broadcast these rules to all the users. This distribution of risk estimation model resembles a decentralised application software in which the application software resides on the user's smartphone. This is in contrast to a centralised software application in which the user sends the query to a centralised server, thereby increasing the risks of information leaks.
- **The communication protocol used to exchange risk messages**: Network protocols lie on a spectrum of centralisation and decentralisation, with privacy-preserving decentralised protocols being preferable for preserving individual rights for contact tracing applications. However, centralisation can be useful for healthcare authorities to monitor the spread of disease and allocate resources.

  The choice of the **communication protocol** used to send risk messages has many implications for user privacy. For a detailed discussion of the pros and cons of various choices, please see section 2 of [1]. Here, we will provide a simplified description of the communication protocol relying on two components. First, we must establish a **contact protocol** that enables two smartphones, when brought into close proximity, to allow a future exchange of risk messages. Second, an **update protocol** that enables the secure transmission of updated risk estimates between these devices. We now present a centralised and decentralised design for each protocol.

The **contact protocol** can be implemented in two ways -

- **A more decentralised contact protocol**, such as that used in GAEN, has two smartphone application users exchange an identifier when they come in close proximity. This is enabled via Bluetooth Low-Energy (BLE) devices. These identifiers are cycled frequently (on the order of 15 minutes) and are only transmitted to nearby devices through Bluetooth.
- **A more centralised contact protocol** may rely on each application to record the GPS coordinates at every fixed interval (e.g., 5 minutes). These coordinates are sent to a central server at a predetermined interval (e.g., 6 hours). The central server runs a matching algorithm that takes in as input the GPS coordinates of all the users and determines which were in close proximity.

The ultimate aim of the **contact protocol** is to generate an identifier or a key (e.g., hash) that can enable future communication between these matched smartphones. The **update protocol** leverages information exchanged during the contact protocol to inform the users of new risk estimates based on their past encounters. The **update protocol** can be more or less centralised. For example -

- **Some more decentralised update protocols** distribute a database of identifiers to all user devices. Each device searches this database and extracts risk messages using identifiers corresponding to past encounters exchanged during the **contact protocol**. Examples of this protocol are Google/Apple Exposure Notification system (GAEN) and Temporary Contact Number (TCN). Please refer to sections 2.3.1 and 2.3.2 of [1].
- **A more centralised update protocol** to send risk messages may rely on the user to send the identifiers received during the contact protocol to the central server. This server returns the risk messages associated with these identifiers, allowing a centralised authority to gather statistics about the distribution of risks and contacts. An example of this protocol is NHS Bluetooth + mix nets, although the software code supports a decentralised version as well. Please refer to section 2.3.3 of [1].

We leave the discussion around the privacy implications of the above choices to [1] (please see section 2).

References

1. Alsdurf H, Belliveau E, Bengio Y, Deleu T, Gupta P, Ippolito D, et al.. COVI White Paper; 2020.
