# Supplementary material for: Proactive Contact Tracing
Source: PLOS Digit Health. 2023 Mar 13;2(3):e0000199. doi: 10.1371/journal.pdig.0000199 (PMC10010527; doi:10.1371/journal.pdig.0000199)
Supplement: S2 Appendix — Further details of COVI-AgentSim. (PDF) [file pdig.0000199.s002.pdf]

S1 Appendix: Agent-based model

Demographics. We sample agent demographics corresponding to the region of Montréal using the census data from Statistics Canada [1]. Specifically, we consider age distribution, statistics on house sizes ranging from 1 to 5, distribution of individual age living alone, and for house sizes ranging from 2 to 5 distribution of the following dwelling characteristics - (a) couple with x kids, (b) single parent with x kids, and (c) random allocation, where x represents number of kids required to complete the house size[1]. Finally, we also consider senior residencies where a proportion of agents above age 65 live. We inform this proportion from the census data as well[2].

The prevalence of selected medical conditions considered as risk factors for COVID-19 disease and severe disease progression were derived from nationally representative surveys and medical surveillance programs in Canada. The following conditions were considered, each with corresponding data sources: heart disease [2–5], stroke [4], asthma [3,4], chronic obstructive pulmonary disease (COPD) [3–5], cancer [3,4,6], diabetes [3–5,7], obesity [3–5,8,9], chronic kidney disease (CKD) [3–5,10], immuno-suppressed conditions [4] and smoking [11,12]. Sex- and age-specific (according to ten year intervals) prevalence estimates were used where data was available. Estimates of age-stratified asymptomatic proportion were obtained from [13].

Mobility. Given an agent population with a designated housing and a workplace (schools for younger population), we assign a mobility schedule to each agent which takes them from one location to another. We use miscellaneous locations to emulate random and relatively shorter interactions typical of dynamics at stores, parks, or gyms. Out of the three broad types of locations, agents spend the majority of their time at their house and workplace. The scheduler is designed to respect constraints, such as, agents younger than 15 years of age are never alone at home, or schedules are dynamically altered to cancel an activity in the event of sickness. We used Statistics Canada data to derive the amount of time spent in each activity and the frequency at which they occur (e.g., every three days). Note that due to the complexities involved in modeling the public transport systems (e.g., large-scale routing and scheduling), our current model lacks such sophistication and, therefore, only accounts for infections happening at locations rather than in the transits.

While the presence of agents at house, workplace, and school is deterministic, to schedule activities like going to cafes, grocery stores, or gyms (collectively termed as random locations) we resorted to probabilistic sampling. Specifically, we used the data for Canada-wide population from Statista and Statscan to inform number of days in which humans are likely to repeat their visit to these random locations. This resulted in mean daily contacts per age group at various locations shown in Figure 1.

Contact Patterns. We implement age-stratified contact sampling. Specifically, for each agent we draw number of contacts as per the location-specific age-stratified number of contacts obtained from the contact matrices. We consider the age groups of 5 years. The contact matrices are derived by projecting empirically derived matrix for Canada [16] on the region of Montréal using demographic standardization [17] and further adjustment to the reported regional mean contacts E_l (see Table 1). Thus, a cell i, j in the matrix denote mean daily contacts made by the agents in age group i with agents in age group j. We use a negative binomial distribution [18] to draw the

[1] We refer the reader to `https://github.com/mila-iqia/COVI-AgentSim/blob/master/src/covid19sim/configs/simulation/region/montreal.yaml` for a fully referenced source of the above parameters.

[2] `https://www12.statcan.gc.ca/census-recensement/2016/dp-pd/covid19/table2-eng.cfm?geo=A0002&S=1&O=A`

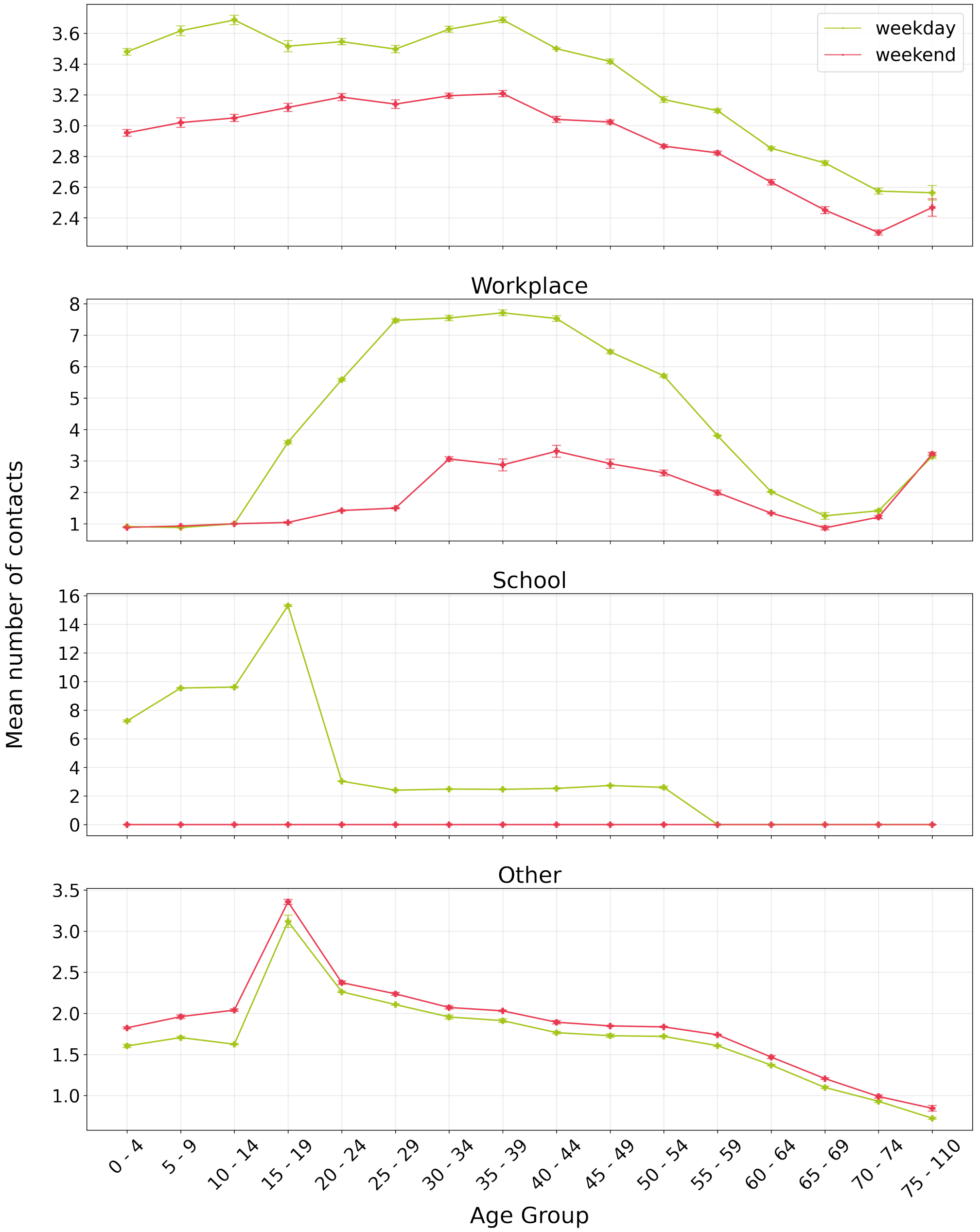

Fig 1. Simulated mean daily contacts on weekdays and weekends broken down by age groups. Agent activities are scheduled such that the mean number of contacts on work and non-work days follow surveyed data as reported in [14, 15]

number of contacts with mean as inferred from the contact matrix and the probability of success as 0.5. Further, we use these matrices to infer probability of interaction with other agents in each age group, thereby, implementing location dependent assortativity in interactions.

The simulated contact patterns with no infections are shown in Figure 2 for overall contacts aggregated across all types of locations. These were obtained by aggregating contacts across 6 simulations with no initial infections, thereby resulting in pre-pandemic contact patterns.

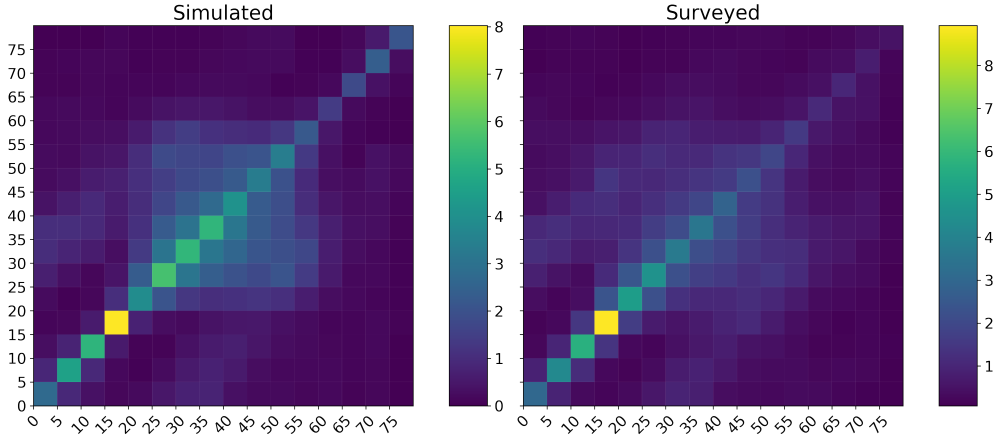

Fig 2. Contact Matrices for all locations: Overall simulated contact pattern (left) yield a similar pattern to empirically derived matrix (right).

Similarly, Figure 3 shows simulated contact patterns for contacts at houses. As a result of housing allocation discussed above, we make two observations (a) there is an oversampling of contacts towards the older age groups: It is because older agents grouped in collectives like senior residencies are modelled explicitly. This choice was motivated from [19] which suggests inclusion of collectives in proper response to the COVID-19 pandemic, (b) a slight discrepancy we observe in the intensity of the main diagonal is due to insufficient social gatherings at households.

Further, Figure 4 shows contact patterns for contacts at schools and workplaces, while the Figure 5 shows contact patterns for contacts at miscellaneous locations. Finally, Figure 6 shows the distribution of mean daily contacts across a population of 10000 agents in a typical simulation without any infection. As expected, we see the presence of agents who sample disproportionately more number of contacts than the average population.

Behavior modification in intermediate behavior levels Table 1 summarizes the location dependent reduction factor α_l for various levels. For the sake of simplicity, we restrict agent behavior levels to one of the top four levels at any point in time. Our choice of using four recommendation levels is motivated by the four-tier measures adopted by Quebec's government[3], though the method is compatible with any number of recommendation levels.

We scale the age-stratified contact matrices with the reduction factor corresponding to the agent's behavior level. Note that in non-PCT scenarios, agents are either in level 1 or level 4. PCT, on the other hand, puts each agent in one of the four levels, based on their estimated risk of infectiousness, mimicking the effect of cautionary behavior

[3] `https://www.quebec.ca/en/health/health-issues/a-z/2019-coronavirus/`

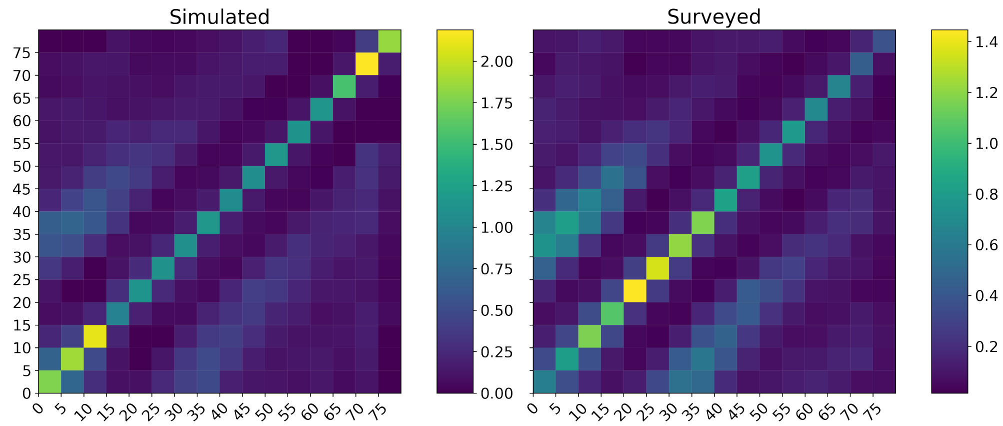

Fig 3. Contact Matrices for House: Housing allocation of agents is calibrated to yield a contact pattern (left) similar to the empirically derived household contact matrices (right). We explicitly model older adults living in assisted care resulting in oversampling of contacts in that age group.

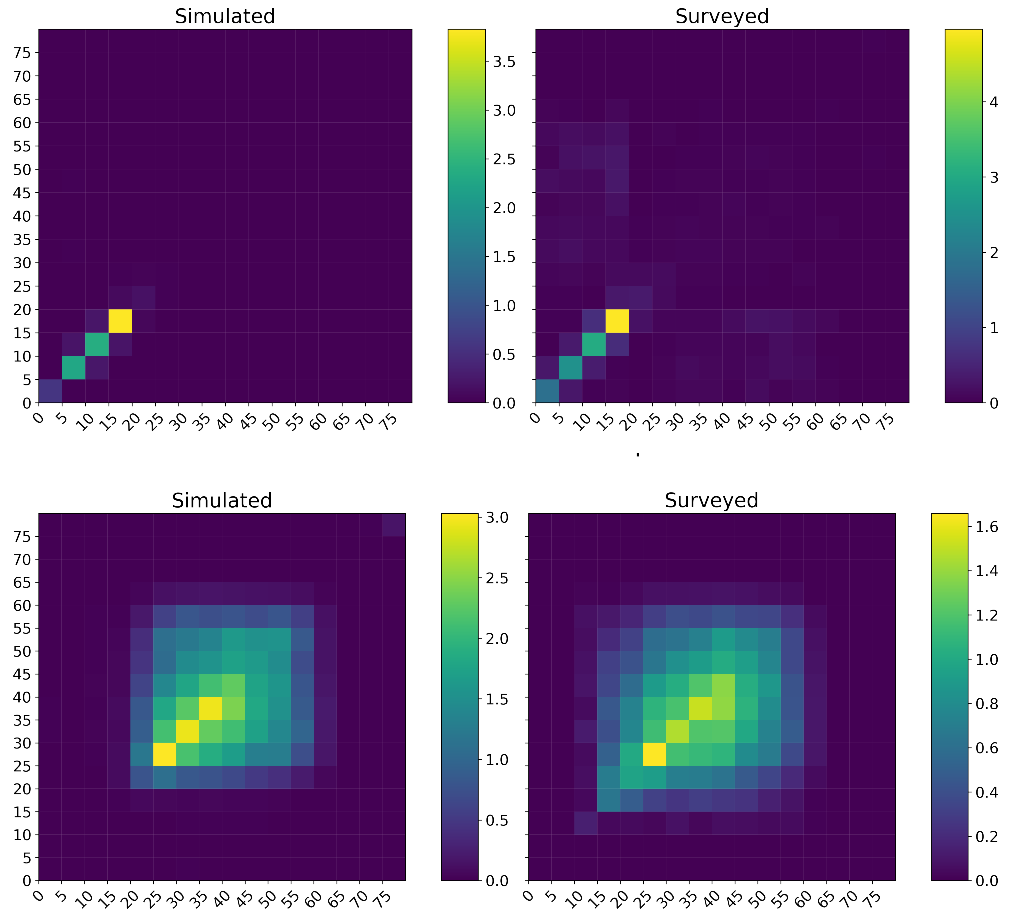

Fig 4. Contact Matrices for School (top) and Workplaces (bottom): Simulated contact pattern at schools (left) yield a similar pattern to empirically derived matrix (right).

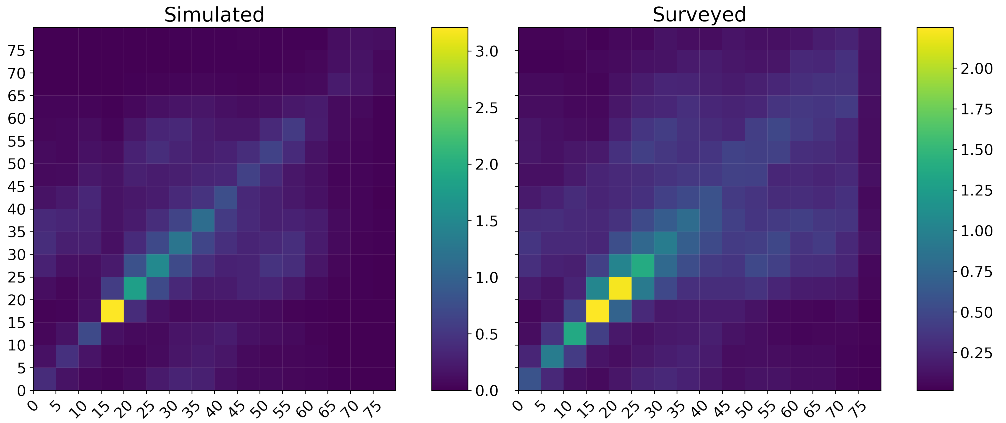

Fig 5. Contact Matrices for "other" locations: Simulated contact pattern at "Other" locations (left) yield a similar pattern to empirically derived matrix (right). Agents spend relatively shorter duration of time at these locations as compared to house or workplaces. Examples of such locations include parks, restaurants, and grocery stores. We observe a slight oversampling of such contacts due to improper estimates of time spent at such activities.

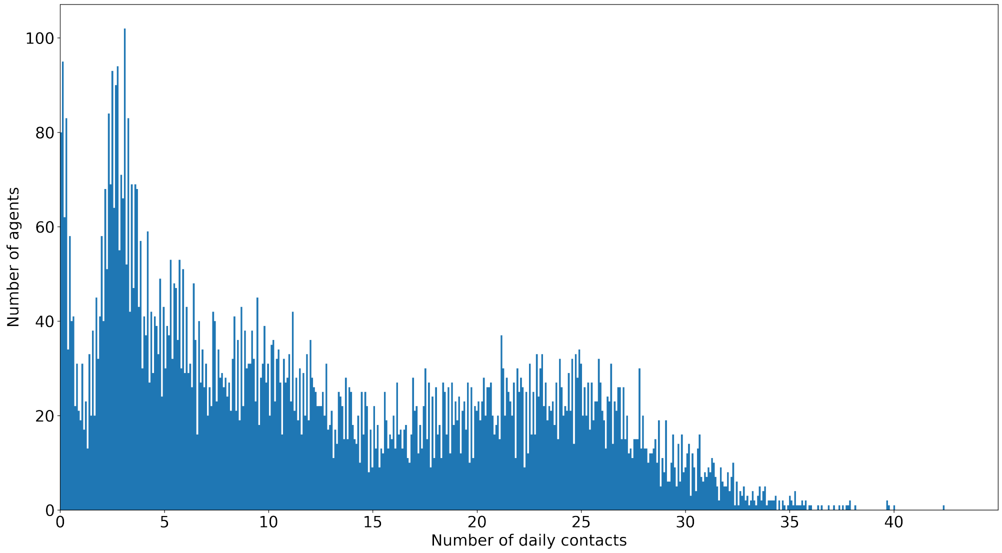

Fig 6. Distribution of daily contacts: The simulation was run with 10000 agents over a period of 30 days with no infection. As an emerging behavior, we observe the agents which sample too many contacts as compared to the average number of contacts (12.62).

Table 1. Daily pre-confinement mean contacts E_l, and reduction factor α_l per location type.

Location	E_l	Level 1 α_l	Level 2 α_l	Level 3 α_l	Level 4 α_l
Household	2.7	0.0675	0.135	0.27	1
Workplace	10	0.18	0.36	0.72	1
School	6	0.18	0.36	0.72	1
Other	3.1	0.1125	0.225	0.45	1

This table shows for each location l the pre-confinement mean number of daily contacts E_l and the reduction factor for the number of contacts used in the simulator. Note that the factor for level 3 is based on data collected in the Region of Montréal [20]

reinforcement via recommendations as experimented by [21]. The resulting pre-pandemic contact patterns are compared to the empirically derived matrices in the supplement. The mean number of contacts, sampled by age group, location type, and day of the week, demonstrate similar trends as reported in the BBC Pandemic Project [14], a recent POLYMOD matrix-based study conducted using smartphone apps.

User compliance to the recommendations is unarguably the most important factor determining the effectiveness of any contact tracing method. For the sake of simplicity, in our simulations, varying levels of behavior restrictions are modeled by a fractional reduction relative to the pre-pandemic number of contacts. However, in its real-life implementation, the app would recommend personalized behaviours (e.g. "avoid public transportation"). These need to be deemed acceptable by app users to achieve high adherence and actualize such reduction in contacts. We direct the readers to the guidelines in [22] for designing such recommendations in close collaboration with user-behavior researchers.

COVID-19 Transmission Model The transmission probability is modeled according to [23], in which the likelihood of transmission is proportional to age-dependent susceptibility (S_a) of the susceptible agent at age a. The probability of transmission also depends on a location-dependent multiplicative factor B_n (for location n), a symptom status (asymptomatic, mild, severe) dependent ratio (A_s) of the infectious agent, and Effective Viral Load, a surrogate for the cumulative viral load (EVL), transmitted from the infectious agent for the duration of exposure time (δt). A multiplicative factor (r) is finally used to calibrate the reproductive number of the infection spread. The formula for the transmission model is presented in Equation 1 and Equation 2 below.

$$\lambda(\delta t, S_a, A_s, n) = \frac{r S_a A_s B_n}{\bar{I}} \int_{\delta t} EVL, \tag{1}$$

$$P(\delta t, S_a, A_s, n) = 1 - e^{-\lambda(\delta t, S_a, A_s, n)}, \tag{2}$$

where $P(\delta t, S_a, A_s, n)$ is the probability of the contagion event. The values for the above constants are directly used from the open source code[4] of [23] .

Effective Viral Load We sample parameters for a piece-wise linear model of what we call **effective viral load** (EVL)[5]. The attributes of EVL are sampled for each

[4] `https://github.com/BDI-pathogens/OpenABM-Covid19/blob/master/documentation/parameters/infection_parameters.md`

[5] Viral load is the number of actual viral RNA in a person; we model a number between 0-1 which could be converted to an actual viral load via multiplying by the maximum amount of viral RNA detectable by a given test.

individual separately. Figure **??** shows a typical shape of EVL motivated from the results in [24], and the attributes' mean and standard deviation follow from [25–27].

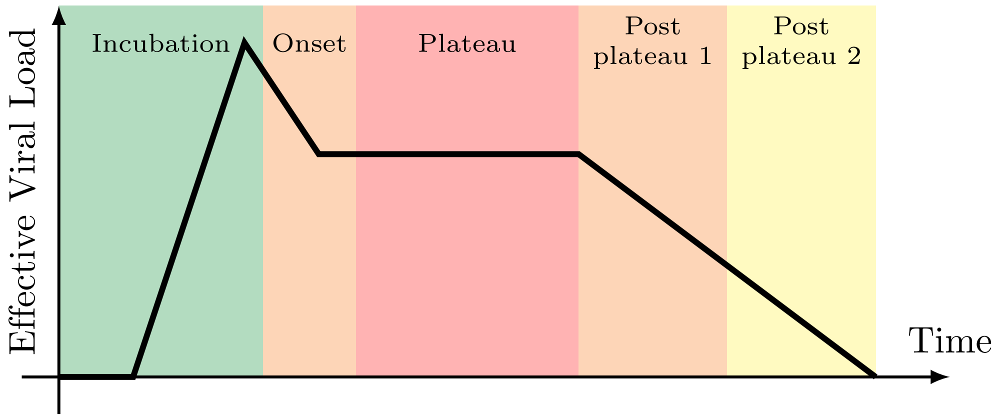

Fig 7. Schematic showing the viral load curve, and associated phases of symptoms with severity indicators: infectiousness onset occurs on average 2.5 days after exposure, viral load peaks 0.7 days before symptom onset, which occurs an average of 5 **incubation days** after exposure [25]. Symptoms are most severe after viral load peak and symptom onset, when the virus has had time to infect many cells. Recovery takes on average 14 days from symptom onset. We use a truncated normal distribution to sample the parameters with the above specified mean values.

Figure 8 is the mean of sampled effective viral load curve. We integrate over individual viral load curve to determine the likelihood of transmission $\lambda(\delta t, S_a, A_s, n)$ as described in Equation 1.

Real-time Polymerase Chain Reaction (RT-PCR) Testing We model the number of available tests as a scarce resource with a fixed number of tests available on any given day, with a delay of two days between testing and when the agent receives the result. These choices are motivated by the challenges in obtaining COVID-19 diagnosis at the pandemic's onset in March 2020. We use a triaging mechanism for tests; an agent experiencing severe symptoms is prioritized over the agents estimated to be in level 4 by a tracing app. Because of diagnostic testing's known performance challenges, a SARS-CoV-2 infection phase-dependent false-negative rate is applied for the RT-PCR results [28].

When an agent gets an RT-PCR test, the agent, as well as other household members, are put into level 4 either (1) until the time of the result (two days) for a negative test result; or (2) for 14 days from the day of the test result for a positive result. An agent with an app can choose to report their test result triggering a notification to their digital contacts. In BCT, such a traced contact and its household members are automatically quarantined for 14 days unless they test negative. To achieve a similar effect of household quarantining in PCT, we require agents to assume the residents' maximum behavior level.

Imperfect agent behavior We use the dropout probabilities as used by [23], such that an agent recommended to quarantine will be 0.01 likely to dropout to the level 1 and sample contacts as per that level. Further, the simulator does not assume all personal information (i.e., age, sex, and pre-existing conditions) will be entered or logged correctly when downloading an app. Instead, the simulator assumes a 70% likelihood that the agent will log their characteristics correctly. Also, an agent is

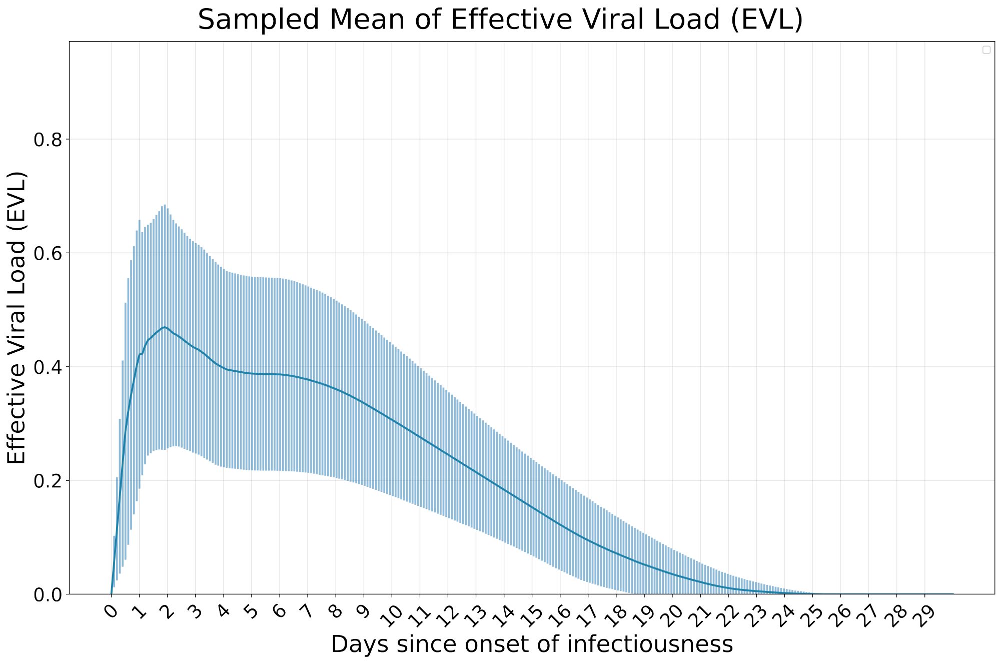

Fig 8. Mean ± 95% C.I of sampled effective viral load of 40 agents.

assumed to be 80% likely to report their RT-PCR test results if they receive a positive test. Imperfect self-reporting of daily symptoms is modeled via two parameters (i) drop-in - the probability of falsely reporting symptoms (to account for malicious behavior), and (ii) dropout - the probability of not reporting a symptom.

App adoption To distribute apps throughout our simulated population, we use an age-dependent distribution across smartphone owners. We use an age-based breakdown of smartphone users as in [23], and use an $UPTAKE$ parameter to vary the population-level adoption rate.

Digital Communication PCT sends risk messages assuming the COVI network communication protocol [22]. This protocol is designed to conform to strict privacy constraints in the following manner: (a) to prevent identification of users, a unique key is exchanged every time two users are within 2 meters of each other for at least 15 minutes; the unique key is then used as a communication channel to exchange risk messages in the future, and (b) risk messages can only carry N bits, constraining their values to one of 2^N values, dramatically reducing the likelihood of de-anonymization. In our experiments, we use $N = 4$ meaning that apps can send risk messages containing a non-negative integer less than 16. As the risk messages form both the input as well as the output of the predictor, we note that the proposed tracing framework is implicitly recursive (i.e. infectiousness of a user is indirectly influenced by all other users in the network).

References

1. Census Profile, 2016 Census - Montréal, Québec and Canada; 2016. Available from: `https://www12.statcan.gc.ca/census-recensement/2016/dp-pd/prof/details/`

page.cfm?Lang=E&Geo1=CMACA&Code1=462&Geo2=PR&Code2=01&Data=Count&SearchText=montreal&SearchType=Begins&SearchPR=01&B1=All&TABID=1.

2. Canada PHAo. type [; 2018]Available from: https://www.canada.ca/en/public-health/services/publications/diseases-conditions/report-heart-disease-Canada-2018.html#a2.
3. Petrilli CM, Jones SA, Yang J, Rajagopalan H, O'Donnell L, Chernyak Y, et al. Factors associated with hospital admission and critical illness among 5279 people with coronavirus disease 2019 in New York City: prospective cohort study. BMJ. 2020;369. doi:10.1136/bmj.m1966.
4. Williamson EJ, Walker AJ, Bhaskaran K, Bacon S, Bates C, Morton CE, et al. Factors associated with COVID-19-related death using OpenSAFELY. Nature. 2020;584(7821):430–436.
5. Killerby ME, Link-Gelles R, Haight SC, Schrodt CA, England L, Gomes DJ, et al. Characteristics associated with hospitalization among patients with COVID-19—Metropolitan Atlanta, Georgia, March–April 2020. Morbidity and Mortality Weekly Report. 2020;69(25):790.
6. Robilotti EV, Babady NE, Mead PA, Rolling T, Perez-Johnston R, Bernardes M, et al. Determinants of COVID-19 disease severity in patients with cancer. Nature medicine. 2020;26(8):1218–1223.
7. Canada PHAo. type [; 2017]Available from: https://www.canada.ca/en/public-health/services/publications/diseases-conditions/diabetes-canada-highlights-chronic-disease-surveillance-system.html.
8. Bello-Chavolla OY, Bahena-López JP, Antonio-Villa NE, Vargas-Vázquez A, González-Díaz A, Márquez-Salinas A, et al. Predicting Mortality Due to SARS-CoV-2: A Mechanistic Score Relating Obesity and Diabetes to COVID-19 Outcomes in Mexico. The Journal of Clinical Endocrinology & Metabolism. 2020;105(8):2752–2761. doi:10.1210/clinem/dgaa346.
9. Overweight and obesity based on measured body mass index, by age group and sex; 2017. Available from: https://www150.statcan.gc.ca/t1/tbl1/en/tv.action?pid=1310037301.
10. Arora P, Vasa P, Brenner D, Iglar K, McFarlane P, Morrison H, et al. Prevalence estimates of chronic kidney disease in Canada: results of a nationally representative survey. CMAJ. 2013;185(9):E417–E423. doi:10.1503/cmaj.120833.
11. Smoking, 2018; 2019. Available from: https://www150.statcan.gc.ca/n1/pub/82-625-x/2019001/article/00006-eng.htm.
12. Organization WH. Smoking and COVID-19; 2020. Available from: https://www.who.int/news-room/commentaries/detail/smoking-and-COVID-19.
13. Davies NG, Klepac P, Liu Y, Prem K, Jit M, Eggo RM. Age-dependent effects in the transmission and control of COVID-19 epidemics. Nature medicine. 2020;26(8):1205–1211.
14. Klepac P, Kucharski AJ, Conlan AJ, Kissler S, Tang M, Fry H, et al. Contacts in context: large-scale setting-specific social mixing matrices from the BBC Pandemic project. medRxiv. 2020;.

15. Mossong J, Hens N, Jit M, Beutels P, Auranen K, Mikolajczyk R, et al. Social Contacts and Mixing Patterns Relevant to the Spread of Infectious Diseases. PLOS Medicine. 2008;5(3):1.

16. Prem K, Cook A, Jit M. Projecting social contact matrices in 152 countries using contact surveys and demographic data. PLoS Computational Biology. 2017;13.

17. Arregui S, Aleta A, Sanz J, Moreno Y. Projecting social contact matrices to different demographic structures. PLoS computational biology. 2018;14(12):e1006638.

18. Wikipedia contributors. Negative binomial distribution; 2020. Available from: `https://en.wikipedia.org/wiki/Negative_binomial_distribution`.

19. Zimmerman S, Sloane PD, Katz PR, Kunze M, O'Neil K, Resnick B. The need to include assisted living in responding to the COVID-19 pandemic. Journal of the American Medical Directors Association. 2020;21(5):572–575.

20. Brisson M, D P, D GGP, D MDP, D JFLP, la contribution de l'équipe d'épidémiologistes et statisticiens et modélisateurs mathématiques et étudiants Myrto Mondor M Sc A, et al.. Épidémiologie et modélisation de l'évolution de la COVID-19 au Québec;. Available from: `https://www.inspq.qc.ca/covid-19/donnees/projections/29-juin`.

21. Ayres I, Romano A, Sotis C. How to Make COVID-19 Contact Tracing Apps work: Insights From Behavioral Economics. Available at SSRN 3689805. 2020;.

22. Alsdurf H, Belliveau E, Bengio Y, Deleu T, Gupta P, Ippolito D, et al.. COVI White Paper; 2020.

23. Ferretti L, Wymant C, Kendall M, Zhao L, Nurtay A, Abeler-Dörner L, et al. Quantifying SARS-CoV-2 transmission suggests epidemic control with digital contact tracing. Science. 2020;368(6491).

24. Goyal A, Cardozo-Ojeda EF, Schiffer JT. Potency and timing of antiviral therapy as determinants of duration of SARS CoV-2 shedding and intensity of inflammatory response. medRxiv. 2020;.

25. He X, Lau EHY, Wu P, Deng X, Wang J, Hao X, et al. Temporal dynamics in viral shedding and transmissibility of COVID-19. Nature Medicine. 2020;26(5):672–675.

26. To KKW, Tsang OTY, Leung WS, Tam AR, Wu TC, Lung DC, et al. Temporal profiles of viral load in posterior oropharyngeal saliva samples and serum antibody responses during infection by SARS-CoV-2: an observational cohort study. The Lancet Infectious Diseases. 2020;.

27. Lauer SA, Grantz KH, Bi Q, Jones FK, Zheng Q, Meredith HR, et al. The incubation period of coronavirus disease 2019 (COVID-19) from publicly reported confirmed cases: estimation and application. Annals of internal medicine. 2020;172(9):577–582.

28. Li D, Wang D, Dong J, Wang N, Huang H, Xu H, et al. False-negative results of real-time reverse-transcriptase polymerase chain reaction for severe acute respiratory syndrome coronavirus 2: role of deep-learning-based CT diagnosis and insights from two cases. Korean journal of radiology. 2020;21(4):505–508.
