# Supplementary material for: Proactive Contact Tracing
Source: PLOS Digit Health. 2023 Mar 13;2(3):e0000199. doi: 10.1371/journal.pdig.0000199 (PMC10010527; doi:10.1371/journal.pdig.0000199)
Supplement: S3 Appendix — Algorithmic description of Rule-based PCT. (PDF) [file pdig.0000199.s003.pdf]

S2 Appendix: Technical Details of Rule-Based PCT

The Rule-Based PCT algorithm takes as input several types of features such as symptoms, test results, and contacts with risky individuals. Based on these features, the algorithm produces an estimate of the user's infectiousness on that day and over the previous 13 days. More abstractly, RB-PCT is a heuristic function which maps a two-dimensional integer array representing 14 days of features related to the likelihood of having or contracting Covid into a single-dimensional array of quantized risk recommendation levels.

Useful Notation To provide a formal description of the RB-PCT algorithm, we now define some terms. In this work, we denote the set of days $\{d_1, d_2, ...d_{max}\}$ as D, where d_{max} is 14. We used 4 recommendation levels, where the lowest recommendation level does not reduce the agent's mobility level, and the highest results in the agent quarantining at home.

Agent's adherence to a recommendation level k entails sampling location specific contact patterns according to that level. We denote agent i's behavior level on day d by ζ_d^i such that, $\zeta_d^i \in \{1, 2, 3, 4\}$.

Determination of this recommendation level is done via a risk estimator that uses a rich suite of features to evaluate agent i's risk history on day t. That risk history is denoted by $\mathbf{r}_t^i$, where we constrain risk levels to be non-negative integers with a maximum value of r_{MAX}. Thus, $\mathbf{r}_t^i \in \{0, 1, 2, ...r_{\text{MAX}}\}^{d_{max}}$ We use $r_{t,d}^i$ to denote estimated risk of agent i for day d such that $t - d_{max} < d < t$. If an agent i had an encounter with an agent j on day d such that $t - d < d_{max}$, $r_{t,d}^i$ is sent to the past contacts as a risk message $M_{i,j}^d(t) = r_{t,d}^i$ if j was a contact of i on day d. This determines what information is available to agent's contacts such that they may modify their own behavior or to propagate risk further.

We use $\mathcal{N}(i)$ to denote a set of agent indices that had at least one digitally recorded contact with agent i in the past d_{max} days. Further, we use the colon symbol (:) to iterate through all possibilities for the variable at that position. For example, $M_{i,:}^d(:)$ represents risk messages from agent i to all agents $j \in \mathcal{N}(i)$ for encounter on day d, if there was one. Similarly, $M_{i,:}^:(:)$ represents risk messages sent by agent i to the agents in $\mathcal{N}(i)$ within the past d_{max} days.

We denote agent i's test result on day d by $\text{test}_d^i \in \{+1, 0, -1\}$, where $+1$ denotes a positive test result, -1 denotes a negative test result, and 0 denotes no test was taken. As a simplifying assumption, we assume that a positive test is retained for d_{max} days while a negative test is retained for d_{min} days (we use $d_{min} = 2$ in our experiments). Thus, an agent i getting a negative test result on day d i.e. $\text{test}_d^i = -1$ will have this variable set to -1 for the next two days i.e. $test_{d+1}^i = -1$, and $test_{d+2}^i = -1$, after which, in the absence of any test $test_{d+3}^i = 0$. We further denote a history of test results for d_{max} days in the past by a vector $\mathbf{T}_d^i \in \{+1, 0, -1\}^{d_{max}}$. We refer to the test result on day d' (where $d - d_{max} < d' \leq d$) as $\mathbf{T}_{d,d'}^i = \text{test}_{d'}^i$ as known on day d.

We similarly denote agent i's symptoms for the past d_{max} days by a matrix $\mathbf{S}_d^i$, such that, with $N_{symptoms}$ categories of symptoms, $\mathbf{S}_d^i \in \{0, 1\}^{N_{symptoms} \times d_{max}}$. Additionally, agent i's symptoms on day d', where $d - d_{max} < d' \leq d$, are represented via $\mathbf{S}_{d,d'}$.

Algorithm The *Rule-based PCT* is implemented as Algorithm 1. We denote S_{HIGH} as the row indices in $\mathbf{S}_d^i \in \{0, 1\}^{N_{symptoms} \times d_{max}}$ such that the symptom at those indices are highly informative symptoms. Similarly, we obtain sets of indices S_{MODERATE} and S_{MILD}. We next describe each function of the *Rule-based PCT*.

1. **Compute Risk** calls each of the below functions in order to generate a risk

history over the last d_{max} days and a current recommendation level. It takes in symptom, test, and risk message data, then takes the element-wise maximum over the risk histories associated with these inputs. *Apply Negative Test* and *Handle Recovery* modify the merged risk history or return an alternative (respectively) to this risk history.

2. **Test Results Compute Risk** covers two cases. A positive test result within the last d_{max} days sets the risk over the past d_{max} days to r_{MAX} and ζ_d^i to 3.

3. **Symptoms Compute Risk** determines the risk and recommendation levels due to symptoms. We group symptoms by how informative they are of COVID-19. Highly informative symptoms experienced within the last $d - d_{max}/2$ days sets high risk levels into $\mathbf{r}^i_{d:d-d_{max}/2}$, and ζ_d^i to 3. Moderately informative symptoms yield moderate risk levels, and so on.

4. **Risk Messages Compute Risk** converts received risk messages into a risk history and recommendation level. We group risk messages into high, medium, and low risk groups, assigning corresponding risk levels to the i's risk history between the day of receipt of that class of risk message and the current day.

5. **Handle Recovery** returns the recent risk history to 0 in the absence of recent evidence of COVID-19. If there are no symptoms within the last 7 days, no recent risk messages, and no positive test results, then we set risk for the past $d_{max/2}$ days as 0.

6. **Apply Negative Test** overwrites the element-wise maximum over the risk histories r_d^i by assigning a 0 risk to the agent around the days when a negative test was reported. Precisely, we denote by W a time window, $W \in \mathcal{N}$ and $W < d_{max}$, such that we set a 0 risk for the agent for $W/2$ days around the day on which negative test was reported ($W = 7$ in our experiments).

Please look at our publicly available code (`https://github.com/mila-iqia/COVI-AgentSim/blob/master/src/covid19sim/interventions/tracing.py#L116`), for a more detailed technical description of *Rule-based PCT* algorithm.

Algorithm 1A *Rule-based PCT*

function TestResultsComputeRisk($\mathbf{T}^i_d$)
  $\mathbf{r} \leftarrow \{0\}^{d_{max}}$
  $\zeta \leftarrow 0$
  if $\sum_{d' \in D} \mathbb{1}_{\{\mathbf{T}^i_{d,d'}=+1\}} \geq 1$ **then**
    $\mathbf{r}_{d:d-d_{max}} \leftarrow r_{\text{MAX}}$
    $\zeta \leftarrow 3$
  return $\mathbf{r}$, ζ
function SymptomsComputeRisk($\mathbf{S}^i_d$)
  $\mathbf{r} \leftarrow \{0\}^{d_{max}}$
  $\zeta \leftarrow 0$
  if $\sum_{d' \in D,\, j \in S_{\text{HIGH}}} \mathbf{S}^i_{d,\{j,d'\}} \geq 1$ **then**
    $\mathbf{r}_{d:d-d_{max}/2} \leftarrow r_{\text{HIGH}}$
    $\zeta \leftarrow 3$
  else if $\sum_{d' \in D,\, j \in S_{\text{MOD}}} \mathbf{S}^i_{d,\{j,d'\}} \geq 1$ **then**
    $\mathbf{r}_{d:d-d_{max}/2} \leftarrow r_{\text{MOD}}$
    $\zeta \leftarrow 2$
  else if $\sum_{d' \in D,\, j \in S_{\text{MILD}}} \mathbf{S}^i_{d,\{j,d'\}} \geq 1$ **then**
    $\mathbf{r}_{d:d-d_{max}/2} \leftarrow r_{\text{MILD}}$
    $\zeta \leftarrow 1$
  return $\mathbf{r}$, ζ
function RiskMessagesComputeRisk($M^{:}_{i,:}(:)$)
  $\mathbf{r} \leftarrow \{0\}^{d_{max}}$
  $\zeta \leftarrow 0$
  if $\sum_{j \in \mathcal{N}(i), d' \in D, d''} \mathbb{1}_{\{M^{d'}_{i,j}(d'')=r_{\text{MAX}}\}} \geq 1$ **then**
    $\hat{d} \leftarrow$ Earliest day of receiving r_{MAX}
    $\mathbf{r}_{d:\hat{d}} \leftarrow r_{\text{MODERATE}}$
    $\zeta \leftarrow 2$
  else if $\sum_{j \in \mathcal{N}(i), d' \in D, d''} \mathbb{1}_{\{M^{d'}_{i,j}(d'')=r_{\text{HIGH}}\}} \geq 1$ **then**
    $\hat{d} \leftarrow$ Earliest day of receiving r_{HIGH}
    $\mathbf{r}_{d:\hat{d}} \leftarrow r_{\text{MILD}}$
    $\zeta \leftarrow 1$
  else if $\sum_{j \in \mathcal{N}(i), d' \in D, d''} \mathbb{1}_{\{M^{d'}_{i,j}(d'')=r_{\text{MOD}}\}} \geq 1$ **then**
    $\hat{d} \leftarrow$ Earliest day of receiving r_{MOD}
    $\mathbf{r}_{d:\hat{d}} \leftarrow r_{\text{MILD}}$
    $\zeta \leftarrow 1$
  return $\mathbf{r}$, ζ

Algorithm 1B *Rule-based PCT*

function HandleRecovery($\mathbf{S}^i_d$, $\mathbf{T}^i_d$, $M^{:}_{i,:}(:)$, $\mathbf{r}^i_d$)
  $\text{Rx} \leftarrow 1$
  $D_7 \leftarrow \{d, d-1, ...d-7\}$
  $D_4 \leftarrow \{d, d-1, ...d-4\}$
  $D_1 \leftarrow \{d, d-1\}$
  if $\sum \mathbf{S}^i_{d,\{:,d:d-d_{max}/2\}} \geq 1$ **then**
    $\text{Rx} \leftarrow 0$
  if $\sum_{d' \in D} \mathbb{1}_{\{\mathbf{T}^i_{d,d'}=+1\}} \geq 1$ **then**
    $\text{Rx} \leftarrow 0$
  if $\sum_{j \in \mathcal{N}(i),\, d' \in D,\, d'' \in D_7} \mathbb{1}_{\{M^{d'}_{i,j}(d'')=r_{\text{HIGH}}\}} \geq 1$ **then**
    $\text{Rx} \leftarrow 0$
  else if $\sum_{j \in \mathcal{N}(i),\, d' \in D,\, d'' \in D_4} \mathbb{1}_{\{M^{d'}_{i,j}(d'')=r_{\text{MOD}}\}} \geq 1$ **then**
    $\text{Rx} \leftarrow 0$
  else if $\sum_{j \in \mathcal{N}(i),\, d' \in D,\, d'' \in D_1} \mathbb{1}_{\{M^{d'}_{i,j}(d'')=r_{\text{MILD}}\}} \geq 1$ **then**
    $\text{Rx} \leftarrow 0$
  if $\text{Rx} = 1$ **then**
    $\mathbf{r}^i_{d,d:d-d_{max}/2} \leftarrow 0$
  return $\mathbf{r}^i_d$, Rx
function ApplyNegativeTest($\zeta^i_d, \mathbf{r}^i_d, \mathbf{T}^i_d, W$)
  $d_n \leftarrow$ day of the latest negative test
  $\mathbf{r}^i_{d,\, d_n - W/2\, :\, d_n + W/2} \leftarrow 0$
  if $\mathbf{r}^i_{d,d} = 0$ **then**
    $\zeta^i_d = 0$
  return $\mathbf{r}^i_d$, ζ^i_d
function ComputeRisk($\mathbf{T}^i_d, \mathbf{S}^i_d, M^{:}_{i,:}(:), \mathbf{X}_i, \mathbf{r}^i_{d-1}$)
  $W \leftarrow 8$
  $\mathbf{r}^i_t, \zeta^i_t \leftarrow$ TestResultsComputeRisk($\mathbf{T}^i_d$)
  $\mathbf{r}^i_s, \zeta^i_s \leftarrow$ SymptomsComputeRisk($\mathbf{S}^i_d$)
  $\mathbf{r}^i_m, \zeta^i_m \leftarrow$ RiskMessagesComputeRisk($M^{:}_{i,:}(:)$)
  $\mathbf{r}_r, \text{Rx} \leftarrow$ HandleRecovery($\mathbf{S}^i_d$, $\mathbf{T}^i_d$, $M^{:}_{i,:}(:)$, $\mathbf{r}^i_{d-1}$)
  if $\text{Rx} = 1$ **then**
    return $\mathbf{r}_r$, 0
  $\mathbf{r}_d \leftarrow \max(\mathbf{r}_t, \mathbf{r}_s, \mathbf{r}_m, \mathbf{r}_{d-1})$ ▷ element-wise max
  $\zeta^i_d \leftarrow \max(\zeta_t, \zeta_s, \zeta_m)$
  if $\sum_{d' \in D} \mathbb{1}_{\{\mathbf{T}_{d,d'}=-1\}} \geq 1$ **then**
    $\mathbf{r}^i_d, \zeta^i_d \leftarrow$ ApplyNegativeTest($\zeta^i_d, \mathbf{r}^i_d, \mathbf{T}^i_d, W$)
  return $\mathbf{r}^i_d$, ζ^i_d
