# Supplementary material for: Proactive Contact Tracing
Source: PLOS Digit Health. 2023 Mar 13;2(3):e0000199. doi: 10.1371/journal.pdig.0000199 (PMC10010527; doi:10.1371/journal.pdig.0000199)
Supplement: S4 Appendix — (PDF) [file pdig.0000199.s004.pdf]

S3 Appendix: Other simulation metrics

In this section we present same plots as in the main section, but for the individual metrics, namely, cumulative incidence, $\hat{H}$, effective contacts, $\hat{E}$, and reproduction number R.

1 Cumulative Incidence $\hat{H}$

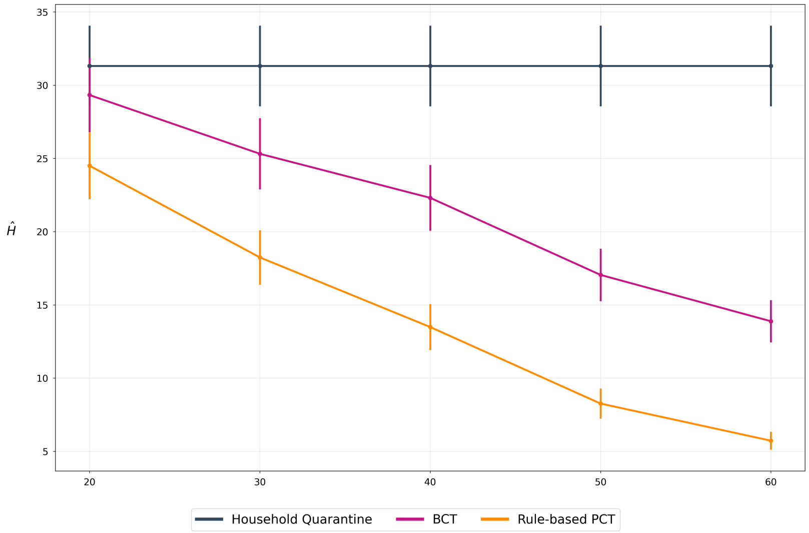

Fig 1. Adoption rate comparison. We compare all methods for adoption rates between 0% (HQ) and 60% of both BCT and PCT.

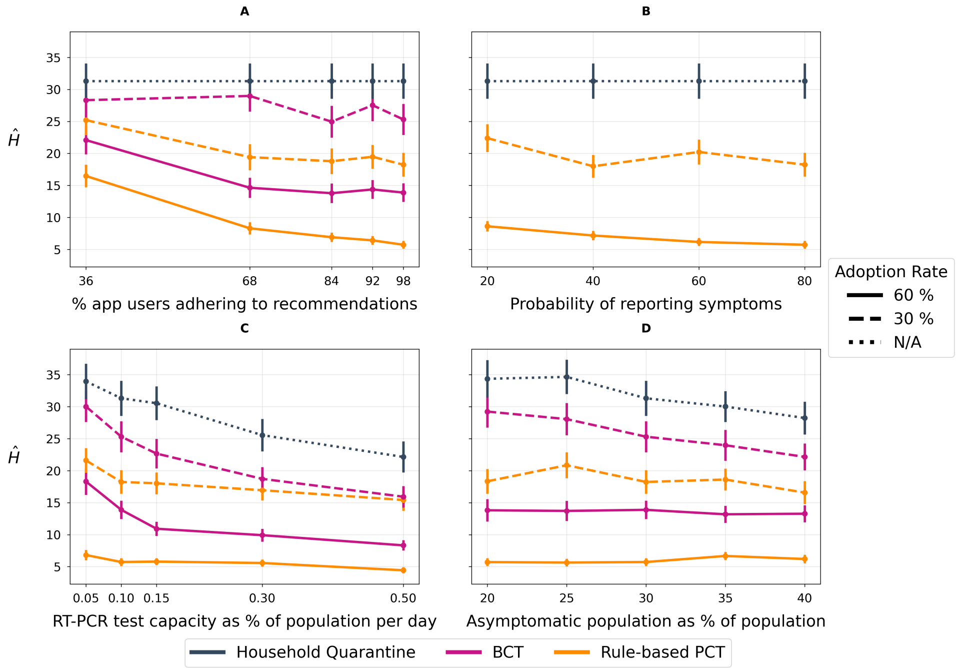

Fig 2. Sensitivity Analyses. We use N/A to represent irrelevance of adoption rate in the baseline scenario as no DCT app is deployed. **(A) Recommendation Adherence.** Illustrates the impact of varying recommendation adherence (e.g. the daily likelihood of getting a test, quarantining, reducing contacts given an in-app notification is received). **(B) Symptom reporting.** Illustrates the impact of varying the daily rate of symptom reporting. Note: the plot omits BCT because BCT doesn't incorporate symptoms in its inputs. **(C) RT-PCR Testing Capacity.** Illustrates the impact of varying the percentage of the population that can receive an RT-PCR test on any given day, ranging from the observed provincial testing capacity of 0.1% to a highly optimistic value of 0.5% of the population. **(D) Infectiousness and symptoms.** Illustrates the impact of varying the proportion of cases that will not develop symptoms.

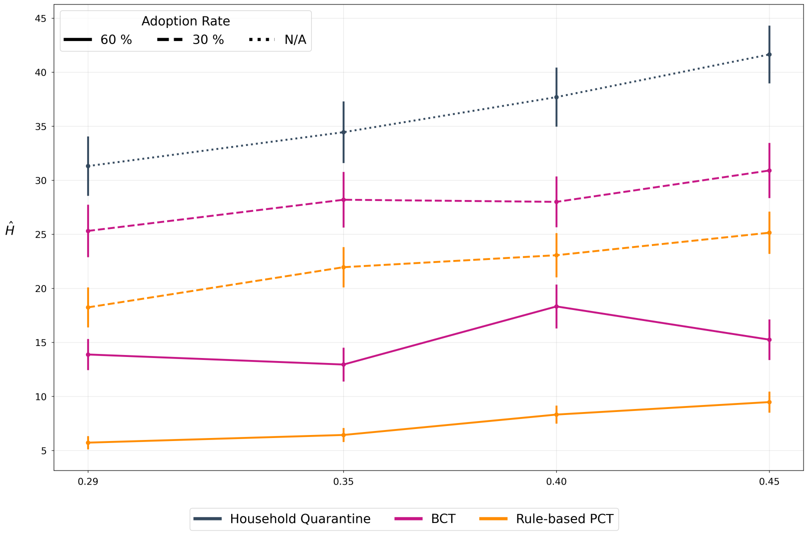

Fig 3. Asymptomatic infection ratio. We vary the relative infectiousness of asymptomatic cases. A value of f implies that the asymptomatic case can potentially infect f times as many people as compared to a symptomatic case. A value of 0.29 is the chosen minimum as described in the epidemiological literature while a higher value of 0.45 is a hypothetical situation describing a more infectious variant of the virus.

2 Effective Contacts $\hat{E}$

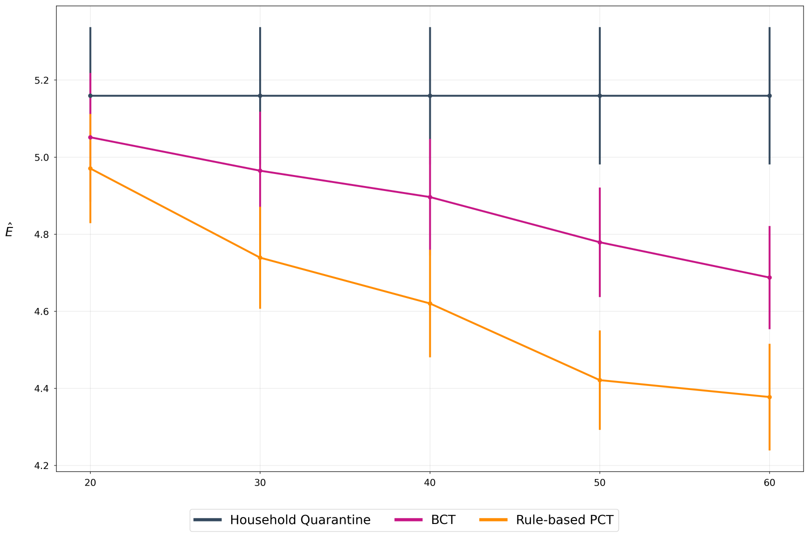

Fig 4. Adoption rate comparison. We compare all methods for adoption rates between 0% (HQ) and 60% of both BCT and PCT.

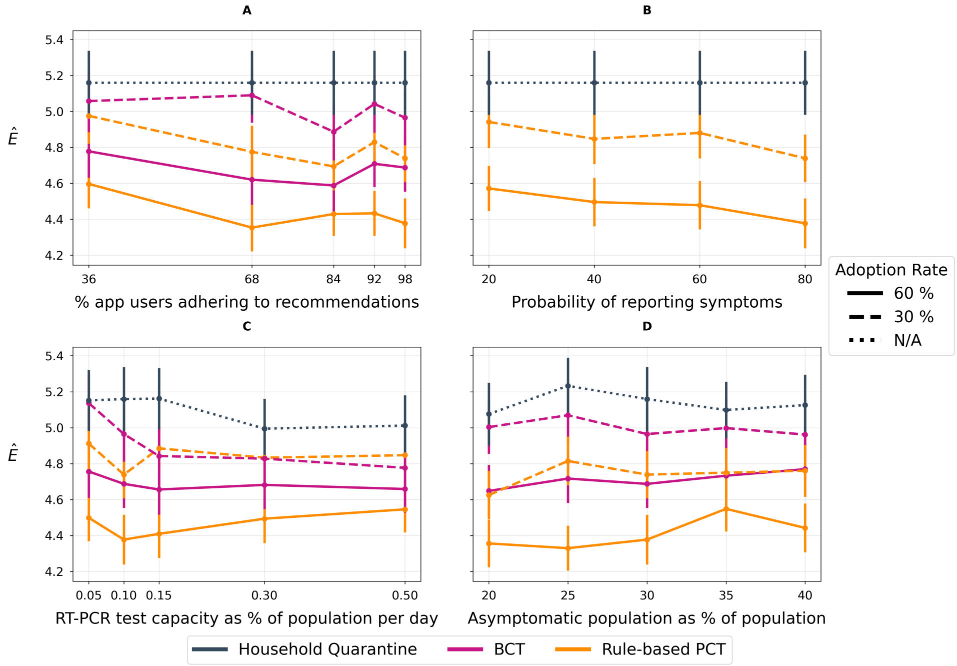

Fig 5. Sensitivity Analyses. We use N/A to represent irrelevance of adoption rate in the baseline scenario as no DCT app is deployed. **(A) Recommendation Adherence.** Illustrates the impact of varying recommendation adherence (e.g. the daily likelihood of getting a test, quarantining, reducing contacts given an in-app notification is received). **(B) Symptom reporting.** Illustrates the impact of varying the daily rate of symptom reporting. Note: the plot omits BCT because BCT doesn't incorporate symptoms in its inputs. **(C) RT-PCR Testing Capacity.** Illustrates the impact of varying the percentage of the population that can receive an RT-PCR test on any given day, ranging from the observed provincial testing capacity of 0.1% to a highly optimistic value of 0.5% of the population. **(D) Infectiousness and symptoms.** Illustrates the impact of varying the proportion of cases that will not develop symptoms.

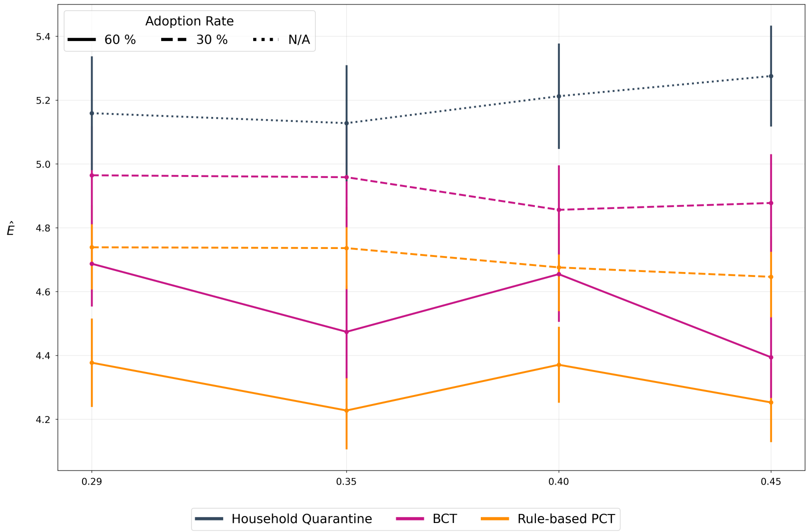

Fig 6. Asymptomatic infection ratio. We vary the relative infectiousness of asymptomatic cases. A value of f implies that the asymptomatic case can potentially infect f times as many people as compared to a symptomatic case. A value of 0.29 is the chosen minimum as described in the epidemiological literature while a higher value of 0.45 is a hypothetical situation describing a more infectious variant of the virus.

3 Reproduction Number R

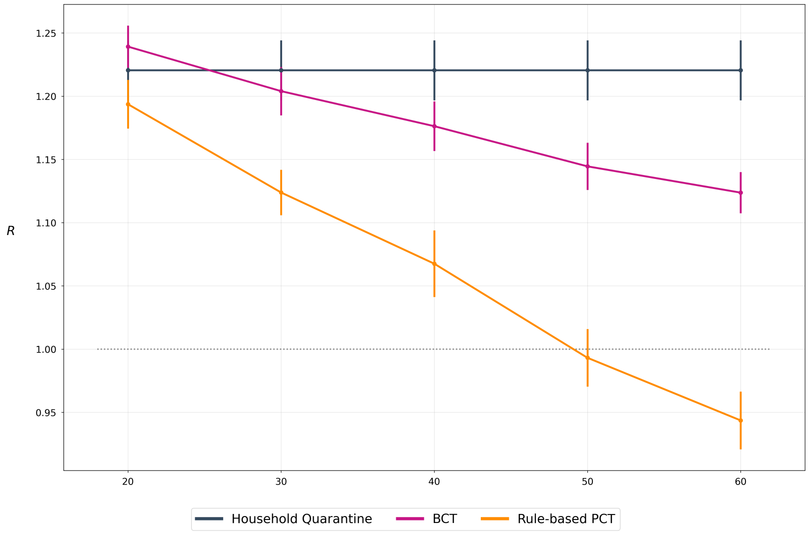

Fig 7. Adoption rate comparison. We compare all methods for adoption rates between 0% (HQ) and 60% of both BCT and PCT.

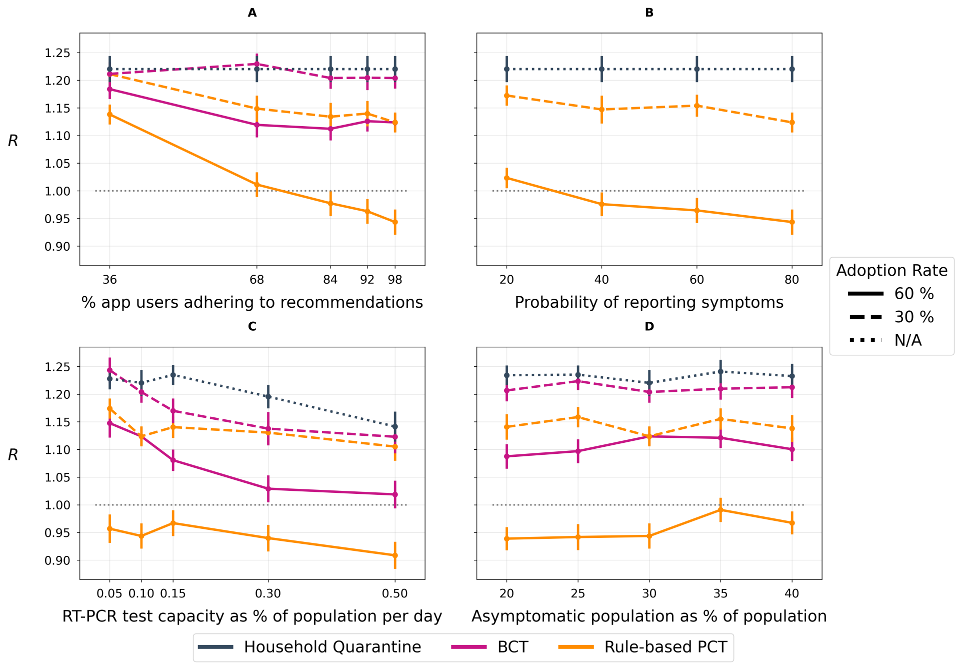

Fig 8. Sensitivity Analyses. We use N/A to represent irrelevance of adoption rate in the baseline scenario as no DCT app is deployed. **(A) Recommendation Adherence.** Illustrates the impact of varying recommendation adherence (e.g. the daily likelihood of getting a test, quarantining, reducing contacts given an in-app notification is received). **(B) Symptom reporting.** Illustrates the impact of varying the daily rate of symptom reporting. Note: the plot omits BCT because BCT doesn't incorporate symptoms in its inputs. **(C) RT-PCR Testing Capacity.** Illustrates the impact of varying the percentage of the population that can receive an RT-PCR test on any given day, ranging from the observed provincial testing capacity of 0.1% to a highly optimistic value of 0.5% of the population. **(D) Infectiousness and symptoms.** Illustrates the impact of varying the proportion of cases that will not develop symptoms.

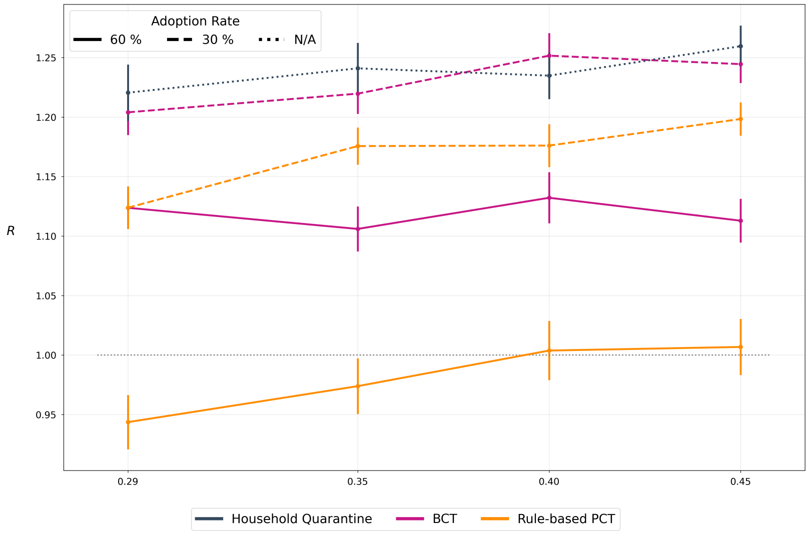

Fig 9. Asymptomatic infection ratio. We vary the relative infectiousness of asymptomatic cases. A value of f implies that the asymptomatic case can potentially infect f times as many people as compared to a symptomatic case. A value of 0.29 is the chosen minimum as described in the epidemiological literature while a higher value of 0.45 is a hypothetical situation describing a more infectious variant of the virus.
