# Supplementary material for: Proactive Contact Tracing
Source: PLOS Digit Health. 2023 Mar 13;2(3):e0000199. doi: 10.1371/journal.pdig.0000199 (PMC10010527; doi:10.1371/journal.pdig.0000199)
Supplement: S5 Appendix — More details on DALYs, TPL, and ICER. (PDF) [file pdig.0000199.s005.pdf]

S4 Appendix: Cost-effectiveness Analysis

DALYs Disability-Adjusted Life Years (DALYs) are a summary measure of the public health burden associated with premature mortality and morbidity due to having a specific disease or health condition. Notably, DALYs are not a direct metric of public health, but rather of the public health *burden*: a decrease in DALYs results in an increase in overall public health. To calculate DALYs, we individually compute the years of life lost due to premature mortality (YLLs) [1] for agents that died during the simulation (i.e. early death), as well as the years of life lost due to disability (YLDs) [2] for agents that were infected and symptomatic. Disability weights (DW) are retrieved from the 2017 Global Burden of Disease Study [3] and represent health preferences such that: DW of 0 is perfect health, and DW of 1 is equivalent to death. DALYs were calculated without discounting or age-weighting, following the methodology set out by the WHO [4]. Life expectancies are retrieved from Statistics Canada [5].

Symptomatic agent status	Disability weight	Equivalent to
Not hospitalized	0.051	Moderate lower respiratory infection
Hospitalized	0.133	Severe lower respiratory infection
Critical care	0.408	Severe COPD without heart failure

Table 1. Disability weights were drawn from the 2017 Global Burden of Disease Study [3]. Since there are no published weights for COVID-19, weights for similar health conditions are selected. As can be seen, higher weights are associated with worse health states. Covid-19 is a novel disease, and there are no published disability weights for different levels of severity of the disease. Therefore, we use similar conditions as proxies for different health states of the agents. Agent hospitalization status is used as a proxy for actual health status, and can be divided into three categories: agents that are symptomatic and not hospitalized, agents that are hospitalized but not in critical care, and agents that are in critical care.

Thus, defining L_i as life expectancy for an agent i,

$$YLL_i = \mathbb{1}_{\text{i died}} \times L_i$$
$$YLD_i = \sum_{s}^{S} DW_s \times l_{i,s}$$
$$DALY_i = YLL_i + YLD_i$$

where:

$$L_i := \text{Life expectancy of agent } i$$
$$DW_s := \text{Disability weight of state } s$$
$$l_{i,s} := \text{duration of state } s \text{ for agent } i$$
$$\mathbb{1}_{\text{i died}} := \begin{cases} 1 & \text{if agent } i \text{ died during the simulation} \\ 0 & \text{otherwise} \end{cases}$$

TPL Temporary Productivity Loss (TPL) is the loss in productivity due to absenteeism from work. To calculate TPL, we extract from the simulator the number of work hours that agents aged 25 to 65 years had to forego due to: (1) quarantine; (2) taking care of a dependent family member (supervision) or; (3) illness that prohibits someone from working. We then multiply this quantity of foregone work hours by the

2019 average hourly wage in Montréal [6] to obtain the total TPL. In computing TPL, we assume that total foregone work hours due to quarantine are scaled by a factor of 0.49 [7] to account for the proportion of agents able to work from home. We follow the methodology for calculating TPL presented in [8].

Aggregated across all agents,

$$n_{25:65} = n_{25:65}^{quarantine} + n_{25:65}^{supervision} + n_{25:65}^{illness}$$
$$TPL = n_{25:65} \times w_{average},$$

where $n_{25:65}^{a}$ is the number of hours foregone due to a, and $w_{average}$ is the average hourly wage for the Montreal workforce.

This approach to evaluating the cost of a healthcare intervention is referred to as the Human Capital Approach (HCA) [8]. The HCA can be used to evaluate the TPL, as well as another form of productivity loss known as the permanent productivity loss due to premature death (PPL) [8]. Given the limited number of agents in our simulator, we avoid calculating the PPL due to the high uncertainty surrounding deaths from COVID19 within the age range of the workforce (25 to 65). Calculating only the TPL instead of both the TPL and the PPL is a more conservative approach: it likely underestimates the impact of our intervention.

ICER

Consider a counterfactual simulation with no pandemic: no DALYs are incurred due to COVID19 and there is no associated TPL. We evaluate different scenarios (HQ, BCT, PCT) by calculating the differences in DALYs and TPL compared to this counterfactual. We then calculate the ICER between each set of two scenarios according to the following formula:

$$ICER = \frac{C_1 - C_0}{E_1 - E_0}$$

where C_i and E_i respectively represent the economic cost (TPL) and health outcomes (avoided DALYs) of intervention i (HQ, BCT or PCT). C_1 and E_1 represent the outcomes of a new intervention that is being considered for adoption, whereas C_0 and E_0 are associated to a *reference intervention*, which is typically a baseline intervention or standard of care [9].

Let us consider how to calculate $E_1 - E_0$: given that we evaluate incurred DALYs compared to a no-pandemic counterfactual with no DALYs lost due to COVID19, the improvement in health outcomes of adopting a new intervention instead of the reference intervention is the *reduction* in DALYs of the new intervention over the reference intervention:

$$E_1 - E_0 = -(DALYs_1 - DALYs_0) = DALYs_0 - DALYs_1$$

Hence, the ICER can be calculated with the following formula:

$$ICER = \frac{TPL_1 - TPL_0}{DALYs_0 - DALYs_1}$$

for a reference intervention indexed by 0 and an alternative intervention indexed by 1.

To calculate the impact of both DCT methods (BCT, PCT) over HQ, we calculate the ICER of each method at various adoption rates with respect to HQ:

$$\frac{TPL_{BCT} - TPL_{\text{HQ}}}{DALYs_{\text{HQ}} - DALYs_{BCT}} \qquad \frac{TPL_{PCT} - TPL_{\text{HQ}}}{DALYs_{\text{HQ}} - DALYs_{PCT}}$$

To further compare between BCT and PCT, we calculate the ICER of PCT with BCT as a reference intervention:

$$\frac{TPL_{PCT} - TPL_{BCT}}{DALYs_{BCT} - DALYs_{PCT}}$$

If the ICER of the new intervention is negative with respect to the reference intervention, then the new intervention is described as "cost-saving" under the assumption that the new intervention results in better health outcomes than the reference intervention ($E_1 > E_0$).

References

1. et al GAR. Global, regional, and national age-sex-specific mortality for 282 causes of death in 195 countries and territories, 1980–2017: a systematic analysis for the Global Burden of Disease Study 2017. The Lancet. 2018;392(10159):1736 – 1788. doi:https://doi.org/10.1016/S0140-6736(18)32203-7.

2. James SL, al. Global, regional, and national incidence, prevalence, and years lived with disability for 354 diseases and injuries for 195 countries and territories, 1990–2017: a systematic analysis for the Global Burden of Disease Study 2017. The Lancet. 2018;392(10159):1789 – 1858. doi:https://doi.org/10.1016/S0140-6736(18)32279-7.

3. of Disease Collaborative Network GB. Global Burden of Disease Study 2017 (GBD 2017) Disability Weights; 2018.

4. Organization WH. WHO methods and data sources for global burden of disease estimates 2000–2016; 2018. Available from: `http://www.who.int/healthinfo/global_burden_disease/GlobalDALY_method_2000_2016.pdf`.

5. Canada S. Table 13-10-0114-01 Life expectancy and other elements of the life table, Canada, all provinces except Prince Edward Island;. Available from: `https://doi.org/10.25318/1310011401-eng`.

6. de la Statistique du Québec I. Weekly and hourly earnings of employees by sex, Montréal and all of Québec, 2015-2019;. Available from: `https://www.stat.gouv.qc.ca/statistiques/profils/profil06/societe/marche_trav/indicat/tra_remuneration06_an.htm`.

7. Gallacher G, Hossain I. Remote Work and Employment Dynamics under Covid-19: Evidence from Canada. Canadian Public Policy. 2020; p. Accepted–version.

8. Nurchis MC, Pascucci D, Sapienza M, Villani L, D'Ambrosio F, Castrini F, et al. Impact of the Burden of COVID-19 in Italy: Results of Disability-Adjusted Life Years (DALYs) and Productivity Loss. International Journal of Environmental Research and Public Health. 2020;17(12):4233.

9. Drummond MF, Sculpher MJ, Claxton K, Stoddart GL, Torrance GW. Methods for the economic evaluation of health care programmes. Oxford university press; 2015.
