# Supplementary material for: Proactive Contact Tracing
Source: PLOS Digit Health. 2023 Mar 13;2(3):e0000199. doi: 10.1371/journal.pdig.0000199 (PMC10010527; doi:10.1371/journal.pdig.0000199)
Supplement: Attachment — Submitted filename: PLOS Response to Reviewers.pdf [file pdig.0000199.s006.pdf]

Dear Editors,

Thank you for giving us the opportunity to submit a revised draft of our manuscript titled "Proactive Contact Tracing" to PLOS Digital Health. We appreciate the time and effort that previous editors and the reviewers have dedicated to providing their valuable feedback on our manuscript. We are grateful to reviewers for their insightful comments on our paper. We have been able to incorporate changes to reflect most of the suggestions provided by the reviewers.

We thank the reviewers for their positive comments on the idea and its presentation. Here, we present our point-by-point comments on each reviewers' concerns.

Reviewer #1

(A) Comment: *"While the increasing prevalence of rapid testing (apparently not modelled for, with RT-PCR testing assumed) may have reduced limitations around waiting for test results somewhat,"*
Response: The simulations have been performed during the early stages of the pandemic when, as evident from the Covid-19 pandemic, the prospects of rapid testing were slim. However, we believe that rapid testing can only prove beneficial to all the tracing methods if the results from rapid testing were to be considered as an input for the apps. There were concerns about the credibility of such tests, thereby making the authorities skeptical of the self-reported tests. We believe that the PCT based apps, through their reliance on multiple sources of information, still have the potential to retain their advantage over the BCT apps. In addition, the rules of the Rule-based PCT algorithm will need modification to reflect a relatively weaker, yet stronger than symptoms, signal than RT-PCR test results.

(B) Comment: *"there remain the usual concerns about its wholly-simulated nature, and whether the proposed model formulation & assumed parameter values (including agent movement, virus transmission dependant on viral load, dropout rate from quarantine, reporting rate, asymptomatic population etc.) indeed reflect real life to sufficient accuracy, including higher-order dynamics."*
Response: We acknowledge this as a valid concern and typical of all simulation-based studies. Simulators can't perfectly imitate the real world. However, we have done our best to inform the parameters of the simulator from the published literature. These parameters are described in the S1 Appendix which has been improved since its previous version (see below for what has been changed). We further provide (in our publicly available code hosted on Github) a well-referenced configuration file that has the values of the parameters used in the simulator.

Further, we want to point out that, even though the current study is focused on comparing the Rule-based PCT algorithm with the baselines, our ideas of transferring the learned algorithm (we allude to this in "Next Steps") to the real world involves iterative learning of the simulator parameters reflective of the real world, thereby ensuring that the predictor learned for PCT is well-suited for the world where it will be deployed. However, this is only possible once we have collected some data after its deployment.

(C) Comment: ***"****five levels of in-app behaviour recommendations are mentioned. However, S1/S4 Appendix appears to mention four recommendation levels. This might be clarified (i.e. is Level 0 considered default/no recommendation? However, it would appear a valid level of agent behaviour within the model?)****"***
Response: We thank the reviewer for bringing this to our attention. We have simplified our description to restrict it to only 4 recommendation levels. Level 0 represents the level of contact before the pandemic. Assuming that two months is too short to have agents fall back to their pre-pandemic behaviour, we haven't considered that to be part of the recommendation system.

(D) Comment: *" it is stated that "...we introduce two intermediate behavior levels {1, 2} each exhibiting <u>double the number of contacts</u> compared to the next higher level". This seems to suggest that with Level 3 (post-confinement behaviour) having x contacts (empirically-determined), Levels 2 and 1 would have 2x and 4x contacts respectively, with Level 0 being the pre-pandemic mean m. However, is it possible that 4x > m, and how were the formulations for 2x and 4x (as opposed to say 1.5x and 2x) determined? This might be briefly discussed."*
Response: We thank the reviewer for pointing this out; the reviewer's logical conclusion will hold. However, the description was not correct. It was meant to be "exhibiting <u>half the *reduction factor*</u> compared to the next higher level". We have made this correction in the current version.

(E) Comment: ***"****The agent-based model appears to sample agent contacts with a common distribution given age status, from its description in the Methods section and S1 Appendix. However, it is not clear whether this adequately models/accounts for outliers, especially individuals/agents that are particularly mobile (i.e. visit many more locations than allowed within the model; this overdispersed nature acknowledged in Line 375). As such, the distribution of daily contacts might also be indicated in Figure 1, with empirical basis if possible.****"***
Response: We thank the reviewer for this comment Our simulator currently doesn't account for agents that visit disproportionately more locations than others. However, we do observe agents that sample disproportionately more contacts than the average. We have provided the figure displaying the distribution of daily contacts (see S1 Appendix Fig 6)

(F) Comment: ***"****Related to the above, it appears that abstract locations (home, workplace, school, etc.) are modelled, instead of specific locations (and [mass public] transportation links between them). It might be clarified as to whether such between-location encounters had been considered within the model."*
Response: Due to the inherent complexity, both in its design as well as the computational overhead in scheduling and planning the routes, we haven't modelled the public-transit system. We have added text clarifying this in the S1 appendix (under the Mobility paragraph).

(G) Comment: "*a global mobility parameter β is introduced and defined, and said to allow the virus containment to be controlled under any scenario by varying the number of daily contacts*

without changing the virus transmission model. However, β does not seem to reappear outside these two sentences. The implementation of β within the model might thus be specified further."
Response: Agree. We have now given an example of how \beta is used in the model. Please see L196.

(H) Comment: "*It might be clarified if repeated contacts with the same (other) agent are considered as separate daily contacts."*
Response: We thank the reviewer for pointing this out. We have now explicitly mentioned how E_hat is computed. It considers the repeated contacts with the same agent as separate contacts.

(I) Comment: "*The performance metric justification might thus be further supported if possible."*
Response: Our criterion for selecting the metric of H/E^2 was merely to bring the metric between 0 and 1. In hindsight, this comes with multiple limitations and was a suboptimal choice. This is also reflected in the comments from other reviewers. We agree that the choice of squaring the denominator reflects a certain preference of the poilicymakers and we, as researchers, do not want to put our own preference over this tradeoff. Therefore, we have now switched our metric of comparison to H/E. We have also provided various other metrics like H, E, and Reproduction Number (R) in S5 and S6 Appendix. We hope that this will clarify the doubts around the metric of comparison.

(J) Comment: "*it is stated that 1000 random subsets of size 60 are drawn from each of 60 simulations for each scenario, to compute the performance. This appears to mean that model standard errors are dependant on the number and size of random subsets chosen. If so, the choice of 1000/60 respectively might be briefly substantiated."*
Response: We chose to run 50 simulations for each scenario. The number of simulations, i.e., 50, was random and merely chosen to fit our experiments within the limited budget of computational resources while obtaining the required statistics for each scenario. However, we take a 1000 random subset with replacement (as in bootstrapping) to compute the standard means and errors of each scenario. We could have chosen to select 5000 random subsets as well. We however don't expect to see a lot of difference because as long as the number of subsets is big enough we would expect the standard means and error to converge to population means and errors.

(K) Comment: *"In the caption for Figure 6, two plausible values (0.29 and 0.45) for the asymptomatic infection ratio are mentioned. However, it is unclear whether the figure describes the various results for these two values. This might be clarified."*
Response: We are sorry that we didn't understand this comment. The sensitivity analysis of asymptomatic infection ratio is done in exactly the same form as the rest of the parameters in Figures 4 and 5.

(L) Comment: *"For the PCT rules in S4 Appendix, the derivation and values of the various parameters might be provided."*
Response: We have now provided more information on the rules and how they were constructed in the S2 Appendix.

Reviewer #2

Comment: *"First, the assessment is done over 2 months. I don't understand why it is so : an epidemic would not last 2 months, and the mobile app would not be discontinued after 2 months. Especially, you show that the number of isolated persons change over time, and it seems that after 2 months at larger adoption rate the number of isolated persons would be larger with PCT than with BCT. I believe that in such a theoretical study, epidemics should be run to their end to compare attack rates and overall impact of social distancing."*
Response: We are sorry that this point was not made clear in our study. We chose the time period of two months as we expect the rules of the Rule-based PCT algorithm to change over time based on the attack rate and government restrictions. We alluded to this point in Conclusion (under Next Steps). Our rules were designed based on the data available at the beginning of the pandemic. Specifically, we think that the continuous recalibration of the simulator and the predictor's retraining will be important to reflect the intricacies in the real-world. See L216-L221 in the main text.

Comment: *"I don't understand the rationale of the metric H/E^2. "*
Response: Please refer to response (I) to the reviewer #1.

Comment: "*the peer-to-peer mechanism critical to the results? would it be the same for a centralised app? I see little evidence to support a particular architecture here; even though it is exepcted that they may have different implications for privacy."*
Response: We thank the reviewer to point this out. Indeed, the peer-to-peer mechanism is not critical. It could also be implemented with a centralized mechanism although with different privacy implications. We have added this discussion in the text as well.

Comment: "*there is no indication of the reproduction ratio and the generation time that is used in the simulations. The construction of EVL is not detailed, but should be. It is said to be piecewise linear but with what parameters? What is the generation time distribution entailed by this curve?"*
Response: We thank the reviewer for this request for clarification. We have now included more details on how EVL is constructed in the S1 Appendix (see Fig. 6 and Fig. 7).

Comment: *"the computation of the contacts is particularly obscure (plus see below for comments about supp1). Are contacts averaged over all individuals over the 2 months?"*

Response: Yes we average contacts across the population and the simulation period. We have provided more details on computing contacts in the main text (under the Evaluation section) as well in the S1 Appendix.

Comment: ***"**Dropout seems very low (probability 0.01 to go to level 1?). Smith (BMJ doi: 10.1136/bmj.n608) reports much larger dropout rates from isolation, with only 40 to 50 % complying overall."*

Response: We are sorry about the confusion. It seems that the dropout parameter was misunderstood. We have conducted sensitivity analysis on dropout values separately in Figure 5(A), where we vary the dropout values from 36% to 98%.

Comment: ***"**P3L12-14 : this is but an exemple. definitions have changed between countries and periods. Insert "for example, in the UK,""*

Response: We have corrected for the language in text now. Please see L12.

Comment: ***"**staying at residence forbids contac with family too?"*

Response: Yes, unless there is a dropout from level 4. This is specified in the last sentence of the paragraph (Please see L136-L139.)

Comment: ***"**I don't see why it must be H/E^2, and not H/E or H/E/10 if you want to keep it between 0 and 1."*

Response: Please refer to (I) in response to reviewer #1.

Comment: **"***This article has not been peer-reviewed. While I agree this is a sentence in the abstract, this conclusion is supported by the cited article."*

Response: Thank you for pointing this out. We have modified the use of this study in our text as suggested by the reviewer.

Comment: ***"**In Table 1, does adoption rate change with age? (independently of owning a mobile phone)**"***

Response: Yes. We follow the same procedure as Ferretti et al. to assume an age-stratified proportion of the population that has smartphones. We model the adoption rate using an uptake parameter that defines the proportion of smartphone users in each age bracket who will adopt the app. We have added this description in the S1 Appendix (see App Adoption paragraph).

Comment: ***"**Sup1 : I have trouble reconciling Fig 1 and Table 1 :……**"***

Response: Excuses. There were mistakes in the compiling of our figures to our manuscript, which lead to the compilation of outdated figures. We have replaced the figures and tables with the most up-to-date and correct figures. In addition, we have added more explanations around parameters in the table.

Comment: ***"**In Fig 5 : is N/A the case of 0% adoption?**"***

Response: N/A is used to say that adoption rate is not applicable as contact tracing application is not being used in the baseline scenario (Household Quarantine). We have added this clarification in the caption of Fig 5.

Reviewer #3

Comment: *"The description of this study was not very informative. I don't see any reason or motivation for this study that needs to be done from the following descriptions written by the authors in Author summary Why Was This Study Done? I suggest the authors improve the writing in this part to highlight their motivation. "*

Response: We thank the reviewer for helping us improve our writing. We looked up more examples from PLOS research articles on how to write this summary. Even though we found that other articles were similarly written than ours, we made some changes as per the suggestions. We hope that this will make the motivation a bit clearer.

Comment: *" In the abstract, "PCT is able to notify potentially infected users as early as possible, preventing onward transmission.". Please clarify "as early as possible"? If PCT allows everyone to be notified, should this be considered as early as possible?"*

Response: We thank the reviewer for pointing this out. It was meant to be a comparative statement implying that PCT is able to notify earlier than BCT. We have made such changes in the abstract now.

Comment: *"In the abstract, "Our results suggest that both BCT and PCT improve upon HQ. However, PCT is more effective than BCT as shown by superior metrics across a range of parameters". What do authors mean by superior metrics?"*

Response: We have replaced this sentence with a more tangible sentence. With efficiency defined in terms of trade-off, we have rephrased the sentence as - *"Our results suggest that both BCT and Rule-based PCT improve upon HQ, however, Rule-based PCT is more efficient at controlling spread of disease than BCT across a range of scenarios."*

Comment: *" In fact, there is not too much description on how this 'Rule-Based' risk assessment is implemented .... This did not give me a clear idea of how they measure the risk. I would say I am really confused about how they estimated the risk based on the symptoms and prior risk history. Could you simply treat a person who has a fever or who is coughing as an infected case and then trace the close contacts? This could cause so many people to be contact-traced during flu seasons. These concerns are important to be clarified."*

Response: We are sorry for the inconvenience. Our detailed description of the rules to measure the risk had been provided in the S2 Appendix. Nevertheless, we have improved our description even further by giving some examples and being more precise about the rules and risk

measurement. Symptoms are only part of the rules and algorithm. Seasonality and the presence of other pathogens leading to the same clinical presentation (respiratory syndrome) will modify the priors and affect the risk.

Comment: *"COVID-19 diagnosis was inefficient because PCR testing was not performed. Therefore, many cases can be wrongly diagnosed or confirmed with delays. If authors would plan to use a symptom-based approach to perform contact-tracing, this is likely to lead to overwhelming of testing, tracing and isolation capacity. Authors should consider this consequence in their modelling."*
Response: We think we might not have fully clarified the role of symptoms in the complete pathway. Our approach is not just symptom-based. PCT aggregates all the signals: test results, symptoms, and risk messages. The aggregation of risk messages is a crucial element as these risk messages are signals of prior infection. How these signals are aggregated is a matter of predictor's design. Depending on the predictor's behaviour, the aforementioned effects can emerge (*overwhelming of testing, tracing and isolation capacity).*

Comment: *"It will be better to show the impact of PCT and BCT on transmissibility (effective reproduction number Re) than Figure 3. It is difficult for me to tell which method (PCT vs BCT) is better in Figure 3. What I suggest is, to calculate the impact on Re at the initial time, peak time, and the late time of the outbreak. Why there are regular fluctuations in simulations should be explained."*
Response: We have now supplied several metrics - H, E, Re in S5 and S6 Appendix. We hope that this will make it clear.

Comment: *" I don't understand equations 1 and 2 in the supplementary file."*
Response: Thanks for pointing this out. We have improved this description.

In addition to the above comments, all spelling and grammatical errors pointed out by the reviewers have been corrected.

We look forward to hearing from you in due time regarding our submission and to responding to any further questions and comments you may have.

Sincerely,
Dr. Yoshua Bengio
