# Supplementary material for: Proactive Contact Tracing
Source: PLOS Digit Health. 2023 Mar 13;2(3):e0000199. doi: 10.1371/journal.pdig.0000199 (PMC10010527; doi:10.1371/journal.pdig.0000199)
Supplement: Attachment — Submitted filename: PLOSDigital Health Rebuttal.pdf [file pdig.0000199.s007.pdf]

Dear Editors,

We highly appreciate the time and effort of both reviewers to provide us with feedback and help us make this submission clear and concise. We thank both reviewers for their positive comments on the idea and its presentation.

In this letter, we present our point-by-point comments on reviewer #2's concerns. We have also changed the manuscript as per the comments. Specifically, we have added an Appendix in response to comment A (with an appropriate reference in the manuscript), and we have changed our discussion in the manuscript according to comment B.

Comment A: *"I'm unable to visualize how PCT would be implemented in a centralized or decentralized DCT architecture…"*

Response:

We thank the reviewer for their question about the communication protocol and associated privacy implications. Here, we will try to address all three clarifying questions brought to our attention in the review.

From a privacy perspective, we can view the architecture of the PCT smartphone application as three components:

1. **On-device execution of the risk estimation model:** A model (e.g., a set of rules) takes in various features as inputs and outputs risk estimates for that user. The features are of two types - (a) locally available on the smartphone, e.g., symptoms, pre-existing conditions, test results, etc., that are used only with the user's consent. This information never leaves the smartphone, mitigating the risk of leaking personally identifiable information. (b) risk messages from past encounters - the smartphone app manages to send and receive these risk messages according to the network protocol. We discuss this in the third point below.

2. **Transmission of the risk estimation model to the device:** The model used for risk estimation is broadcasted to the users at a predetermined frequency. For example, it may be desirable to update the rules every two weeks, in which case a centralised server would broadcast these rules to all the users. This distribution of risk estimation model resembles a decentralised application software in which the application software resides on the user's smartphone. This is in contrast to a

centralised software application in which the user sends the query to a centralised server, thereby increasing the risks of information leaks.

3. **The communication protocol used to exchange risk messages:** Network protocols lie on a spectrum of centralisation and decentralisation, with privacy-preserving decentralised protocols being preferable for preserving individual rights for contact tracing applications. However, centralisation can be useful for healthcare authorities to monitor the spread of disease and allocate resources.

   The choice of the **communication protocol** used to send risk messages has many implications for user privacy. For a detailed discussion of the pros and cons of various choices, please see section 2 of *Alsdurf et al. 2020*. Here, we will provide a simplified description of the communication protocol relying on two components. First, we must establish a **contact protocol** that enables two smartphones, when brought into close proximity, to allow a future exchange of risk messages. Second, an **update protocol** that enables the secure transmission of updated risk estimates between these devices. We now present a centralised and decentralised design for each protocol.

   The **contact protocol** can be implemented in two ways -

   a. **A more decentralised contact protocol,** such as that used in GAEN, has two smartphone application users exchange an identifier when they come in close proximity. This is enabled via Bluetooth Low-Energy (BLE) devices. These identifiers are cycled frequently (on the order of 15 minutes) and are only transmitted to nearby devices through Bluetooth.
   b. **A more centralised contact protocol** may rely on each application to record the GPS coordinates at every fixed interval (e.g., 5 minutes). These coordinates are sent to a central server at a predetermined interval (e.g., 6 hours). The central server runs a matching algorithm that takes in as input the GPS coordinates of all the users and determines which were in close proximity.

   The ultimate aim of the **contact protocol** is to generate an identifier or a key (e.g., hash) that can enable future communication between these matched smartphones. The **update protocol** leverages information exchanged during the contact protocol to inform the users of new risk estimates based on their past encounters. The **update protocol** can be more or less centralised. For example -

a. **Some more decentralised update protocols** distribute a database of identifiers to all user devices. Each device searches this database and extracts risk messages using identifiers corresponding to past encounters exchanged during the **contact protocol**. Examples of this protocol are Google/Apple Exposure Notification system (GAEN) and Temporary Contact Number (TCN). Please refer to sections 2.3.1 and 2.3.2 of Alsdurf et al. 2020.
b. **A more centralised update protocol** to send risk messages may rely on the user to send the identifiers received during the contact protocol to the central server. This server returns the risk messages associated with these identifiers, allowing a centralised authority to gather statistics about the distribution of risks and contacts. An example of this protocol is NHS Bluetooth + mix nets, although the software code supports a decentralised version as well. Please refer to section 2.3.3 of Alsdurf et al. 2020.

We leave the discussion around the privacy implications of the above choices to Alsdurf et al. 2020 (please see section 2).

To respond to the reviewers' sub-comments,

- **Comment A.1.** asks "*how the peer-to-peer [communication] mechanism is not critical to results (as stated in the response to reviewer #2) when peer-to-peer communication seems to be the only way the risk messages would be received in a decentralized DCT system.*" We provide an example of a centralized communication protocol in our point 3 above. Moreover, in our submission, we intend to separate out the privacy implications of various choices from the risk estimation framework.
- **Comment A.2** asks " *how the current and past 14 days infectiousness is estimated privately (line 55)*" - We describe the steps involved in risk estimation through our points 1 and 2 above. We emphasize that individual's symptom and pre-existing condition data is kept private and on-device.
- **Comment A.3** asks "*how the protocol integrates individualized data in a privacy-preserving manner (line 344)*". In addition to the response to Comment A.2 above, we emphasize that PCT only requires risk messages to be communicated between users. With a secure communication protocol, the PCT will preserve the privacy of the risk-levels as well. If the chosen communication protocol is insecure, then only information about user risk level could be leaked.

Reference:
Alsdurf et al. 2020 COVI White Paper - Version 1.1 https://arxiv.org/pdf/2005.08502.pdf

Comment B: “*With regard to the cost effectiveness analysis, the 20K Canadian dollar per DALY averted threshold is out of date. The article referenced is from 1992 and uses 1990 Canadian dollars. The current manuscript doesn't include a PPP adjustment, so it's likely to be an underestimate of current willingness to pay thresholds. The analysis should use a more contemporary threshold for Canada. This will affect the comparison of the ICER of BCT and PCT with the willingness to pay threshold.*”

Response: We appreciate the feedback from the reviewer regarding the concerns about PPP adjustment. The choice of an appropriate willingness-to-pay (WTP) threshold is a challenge in North America, as there is no set agreed-upon standard. In a recent systematic review by McDougall et al. (2020), the published ICER thresholds for Canada referenced were $20,000 CAD, as well as the range of $32,000-$137,000 CAD per QALY. These wide ranges, primarily driven by WTP thresholds from the oncology space, highlight the need for a clearly agreed upon WTP threshold that is applicable to new technologies, such as mobile apps for COVID-19. We have raised this concern in the Limitations section as well (see lines 381-386 in the marked-up manuscript).

However, recognizing that the above threshold of $20K was established in 1992, we have adjusted the threshold based on the Canadian Consumer Price Index (CPI) to adjust for inflation. The new threshold, as adjusted for inflation, is $33K. We have also shown the computation for this adjustment in the S4 appendix.

Finally, as justified in Griffiths et al. (2016), the threshold of $50K CAD per QALY as proposed by the Canadian Agency for Drugs and Technology in Health (CADTH) is neither consistently applied nor explicitly stated. Therefore, we decided to remove this threshold. We have updated the figure and the discussion in the main text accordingly (see Fig. 7(b), lines 347-349 in the marked-up manuscript).

We are glad to have the opportunity to discuss this dilemma. We would appreciate any thoughts of the reviewer in this line of discussion.

References:

Jean A. McDougall, Wesley E. Furnback, Bruce C. M. Wang & Jörg Mahlich (2020). Understanding the global measurement of willingness to pay in health, Journal of Market
Access & Health Policy, 8:1, 1717030, DOI: 10.1080/20016689.2020.1717030

CPI: https://www150.statcan.gc.ca/t1/tbl1/en/tv.action?pid=1810000501

Griffiths, E. A., and Nirma Khatri Vadlamudi. "Cadth’s $50,000 cost-effectiveness threshold: fact or fiction?." *Value in Health* 19, no. 7 (2016): A488-A489.

We hope that our response has clarified the reviewer’s concerns and that changes made to the manuscript will be satisfactory for the reviewers. We look forward to hearing from you in due time to respond to any further questions and comments that the reviewers may have.

Sincerely,
Dr Yoshua Bengio
